# Complete genome sequence of *Bifidobacterium animalis* B01

**DOI:** 10.1128/mra.00394-24

**Published:** 2024-07-05

**Authors:** Jiahao Li, Xiuyun Wu, Bo Ding, Sheng Xing

**Affiliations:** 1 The State Key Laboratory of Microbial Technology, Shandong University, Qingdao, Shandong, China; 2 Shan Dong Institute for Food and Drug Control, Jinan, Shandong, China; 3 NMPA Key Laboratory for Research and Evaluation of Generic Drugs, Jinan, Shandong, China; Portland State University, Portland, Oregon, USA

**Keywords:** genome sequence

## Abstract

*Bifidobacterium*, a class of strictly anaerobic Gram-positive bacteria, existed mainly in the gut of humans and many mammals. Here, we report the complete genome sequence of *Bifidobacterium animalis* B01, whose genome length is 1,935,423 bp with a GC content of 60.49%.

## ANNOUNCEMENT

*Bifidobacterium* is a class of strictly anaerobic Gram-positive bacteria and plays an important role in regulating the balance of intestinal flora, promoting normal intestinal development, anti-inflammation (1 – 3). Genome sequencing and analysis of *Bifidobacterium* could reveal the physiological and metabolic mechanism from the molecular level and explore the important functional genes. *Bifidobacterium animalis* B01 was isolated in March 2023 from a mixed stool sample of healthy children in Jinan, Shandong province, China. After 0.1 g sample was added to 1 mL 0.9% sterile sodium chloride solution, the serially diluted mixtures were coated onto improved TPY plates (Purchased from QingDao Hopebio-Technology Co. Ltd.) and underwent anaerobic incubation at 37°C for 72 h. Single colonies were selected for 16S rRNA amplification by PCR with the primer pair 27F and 1492R. The 16S rRNA gene was sequenced by Sanger dideoxy sequencing and blast with the NCBI Nucleotide collection database. The colony B01 was identified as *B. animalis* with 16S rRNA sequence (GenBank accession number PP542517).

*B. animalis* B01 underwent anaerobic culture in TPY fluid medium at 37°C for 48 h. The genomic DNA was extracted using the QIAGEN Genomic-tip 20/G kit (QIAGEN). Oxford Nanopore Technologies (ONT) and Illumina libraries were prepared from the same DNA preparation. For Illumina sequencing, DNA sample was sheared into 400–500-bp fragments using a Covaris M220 Focused Acoustic Shearer. Then, the library was prepared from the sheared fragments using the TrueLib DNA Library Rapid Prep Kit (300 cycles) and paired-end sequencing (2 × 150 bp) on an Illumina Novaseq 6000 platform. For ONT sequencing, DNA fragments were recovered using BluePippin’s fully automated nucleic acid recovery system and then attached sequencing adapters supplied in the SQK-LSK109 kit to the DNA ends. Then, the library was sequenced on an PromethION48 sequencer with PromethION Flow Cell (R9 Version). Illumina sequencing generated 4,302,275 * 2 raw pair reads, 1,285,425,814 bases, with 97.68% coverage based on K-mer analysis and raw Q30 of 92.73%. The raw reads generated from the paired-end library were subjected to quality filtration using fastp v0.23.0. ONT sequencing generated 100,191 reads, 828,496,073 bases, with 99.47% coverage base on reads comparison and the largest, average length and reads N50 value of 94,182 bp, 8,269.17 bp and 12,036 bp. ONT reads were extracted, base called, demultiplexed, and trimmed with the minimum Q score cutoff of 7. Guppy v5.0.16 was used as the base caller (4). The sequencing depth for Illumina and ONT was 428.07 and 664.16, respectively. Unicycler v0.4.8 (5) was used for hybrid assembly of Illumina data after quality control and ONT data, and Pilon v1.22 (6) was used for correction. Finally, after the overlap sequence at both ends of the assembly sequence was cut off, a complete circular chromosome sequence, that is, the sequence of *B. animalis* B01, was obtained. Genome annotation was performed using the NCBI Prokaryotic Genome Annotation Pipeline (PGAP v5.3) (7). Default parameters were used for all software unless otherwise specified.

The total genome length of *B. animalis* B01 was 1,935,423 bp, containing 1,534 protein-coding genes, 52 tRNAs, 9 rRNAs, with the average GC content of 60.49%.

## Data Availability

The genome sequence of *B. animalis* B01 is available in the NCBI GenBank under genome accession number CP138633. The raw sequence data are available under Sequence Read Archive (SRA) accession numbers SRX22999872 (Illumina) and SRX22999873 (ONT).
